# A novel inflammation-based prognostic score in esophageal squamous cell carcinoma: the C-reactive protein/albumin ratio

**DOI:** 10.1186/s12885-015-1379-6

**Published:** 2015-05-02

**Authors:** Xiao-li Wei, Feng-hua Wang, Dong-sheng Zhang, Miao-zhen Qiu, Chao Ren, Ying Jin, Yi-xin Zhou, De-shen Wang, Ming-ming He, Long Bai, Feng Wang, Hui-yan Luo, Yu-hong Li, Rui-hua Xu

**Affiliations:** Department of Medical Oncology, Sun Yat-sen University Cancer Center, State Key Laboratory of Oncology in South China, Collaborative Innovation Center for Cancer Medicine, 651 Dong Feng Road East, Guangzhou, 510060 Guangdong Province China

**Keywords:** Esophageal squamous cell carcinoma, C-reactive protein, Albumin, The modified Glasgow Prognostic Score, Inflammation-based prognostic score, Survival

## Abstract

**Background:**

Plenty of studies have demonstrated the prognostic value of various inflammation-based indexes in cancer. This study was designed to investigate the prognostic value of the C-reactive protein/albumin (CRP/Alb) ratio in esophageal squamous cell carcinoma.

**Methods:**

A retrospective study of 423 cases with newly diagnosed esophageal squamous cell carcinoma was conducted. We analyzed the association of the CRP/Alb ratio with clinicopathologic characteristics. The prognostic value was explored by univariate and multivariate survival analysis. In addition, we compared the discriminatory ability of the CRP/Alb ratio with other inflammation-based prognostic scores by evaluating the area under the receiver operating characteristics curves (AUC), including the modified Glasgow Prognostic Score (mGPS), neutrophil lymphocyte ratio (NLR) and platelet lymphocyte ratio (PLR).

**Results:**

The optimal cut-off value was identified to be 0.095 for the CRP/Alb ratio. A higher level of the CRP/Alb ratio was associated with larger tumor size (*P* < 0.001), poorer differentiation (*P* = 0.019), deeper tumor invasion (*P* = 0.003), more lymph node metastasis (*P* = 0.015), more distant metastasis (*P* < 0.001) and later TNM stage (*P* < 0.001). The CRP/Alb ratio was identified to be the only inflammation-based prognostic score with independent association with overall survival by multivariate analysis (*P* = 0.031). The AUC value of the CRP/Alb ratio was higher compared with the NLR and PLR, but not mGPS at 6, 12 and 24 months of follow-up. In addition, the CRP/Alb ratio could identify a group of patients with mGPS score of 0 who had comparable overall survival with those with mGPS score of 1.

**Conclusions:**

The CRP/Alb ratio is a novel but promising inflammation-based prognostic score in esophageal squamous cell carcinoma. It is a valuable coadjutant for the mGPS to further identify patients’ survival differences.

**Electronic supplementary material:**

The online version of this article (doi:10.1186/s12885-015-1379-6) contains supplementary material, which is available to authorized users.

## Background

Esophageal cancer (EC) is one of the most common malignancies in the digestive system. The main pathological subtypes include adenocarcinoma and squamous cell carcinoma. Esophageal adenocarcinoma (EAC) is the major subtype in some Western countries [1], while the incidence of esophageal squamous cell carcinoma (ESCC) is higher in some Asian countries, with China included [2,3]. Although great progress has been made in the treatment in recent decades, the prognosis of EC remains poor. The American Joint Committee on Cancer (AJCC) and the Union for International Cancer Control (UICC) tumor-node-metastasis (TNM) staging system is the most important prognostic indicator [4,5]. Recently increasing researches focus on the identification of other promising prognostic factors, and such research achievements, to some degree, may not only contribute to the classification and management of patients in clinical practice, but also facilitate the progress of translational research.

In addition to the histopathological factors and tumor stage, some other prognostic indicators have been discovered by previous studies [6,7]. Nutritional conditions affect patient outcomes with EC to a large extent, partially owning to its anatomic location of upper digestive tract. Besides, the levels of inflammation also play important roles in patient status and tumor progression. Accordingly, nutrition-based and/or inflammation-based prognostic indicators, such as body mass index (BMI) [8], the modified Glasgow Prognostic Score (mGPS) [9], the prognostic nutritional index (PNI) [10], the neutrophil-lymphocyte ratio (NLR) and the platelet-lymphocyte ratio (PLR) [9], have emerged as prognostic factors in EC as well as various other cancers [11-13].

The C-reactive protein albumin (CRP/Alb) ratio, a novel inflammation-based prognostic score, has been demonstrated to show outstanding prognostic value in hepatocellular carcinoma compared with other established inflammation-based prognostic scores [14]. In this study, we aim to explore the prognostic performance of the CRP/Alb ratio in Chinese patients with ESCC, and compare it with other established inflammation-based prognostic scores.

## Methods

### Ethics statement

All patients have provided written informed consent for their information to be stored and used in the hospital database. Study approval was obtained from independent ethics committees at Sun Yat-sen University Cancer Center. This study was conducted in accordance with the ethical standards of the World Medical Association Declaration of Helsinki.

### Study population

We retrospectively reviewed the medical records of 649 cases with newly diagnosed esophageal malignancies from October 1, 2006 to November 30, 2010 in Sun Yat-sen University Cancer Center in Guangzhou, China. Pathological diagnoses were carefully checked. We excluded patients without pathological diagnosis and patients diagnosed with other malignancies, such as EAC, esophageal small cell carcinoma, esophageal carcinosarcoma and so on. Only patients pathologically confirmed with ESCC were enrolled in this study. One patient with cervical cancer diagnosed within five years before the diagnosis of ESCC was also excluded. Moreover, we also excluded patients without pretreatment information of nutrition and/or inflammation-based prognostic indicators, patients lost to follow-up, as well as patients died of non-cancer causes. Furthermore, to eliminate the influences of non-cancer diseases on inflammation-based prognostic scores, we excluded patients with rheumatoid diseases and acute infection. Finally, there were 423 cases enrolled in our study. Clinicopathologic information and pretreatment nutrition and inflammation-based indexes were retrospectively collected.

### Measurement of several tumor-related characteristics

The tumor stage was classified according to the AJCC/UICC TNM staging system (the 7th edition). For the T classification, most of the cases with stage I – III ESCC were classified by post-operative pathological specimens. There were thirteen cases classified as T4b after an exploratory thoracotomy and with unresectable locally invasive tumors founded. The tumor size was defined as the long diameter measured with post-operative pathological specimens. The tumor locations were classified into upper esophagus, middle esophagus and lower esophagus. Because of the small numbers of tumors located in cervical esophagus and gastroesophageal junction, we categorized tumors located in cervical esophagus into the upper esophagus group, and tumors located in gastroesophageal junction into the lower esophagus group in this study.

### Definitions of various nutrition and inflammation-based prognostic scores

The nutrition and inflammation-based prognostic scores in this study were defined and calculated as follows. BMI: body mass index, calculated by weight (Kg)/height (m) ^2^. mGPS: the Glasgow Prognostic Score, it was the combination of CRP and albumin. Patients with CRP < 10 mg/L were allocated a score of 0. Patients with both CRP > 10 mg/L and albumin > 35 g/L were allocated a score of 1. Patients with both CRP > 10 mg/L and albumin < 35 g/L were allocated a score of 2. PNI: the prognostic nutritional index, it was calculated by the formula of 10 × albumin (g/dL) + 0.005× lymphocyte count/uL. NLR: the neutrophil-lymphocyte ratio . PLR: the platelet-lymphocyte ratio. CRP/Alb: the CRP (mg/L)-albumin (g/L) ratio. All the indicators involved in the calculation of the nutrition and inflammation-based prognostic scores were tested before surgery, chemotherapy and radiotherapy treatment. Patients without pretreatment information for these indicators or patients who had received surgery, chemotherapy or radiotherapy in other hospitals before they came to our center were both excluded from this research. Therefore the impact of surgery, chemotherapy and radiotherapy on these scores could be avoided.

### Treatment and follow-up of patients

Patients of all TNM stages were enrolled in this study. The treatment strategies were made according to the National Comprehensive Cancer Network (NCCN) Clinical Practice guidelines. Because patients involved in this study were diagnosed from October 1, 2006 to November 30, 2010, the modality of therapy combination was diversiform. To analyze the impact of treatment on survival of patients, we categorized all the patients into two groups, including curative treatment group and palliative treatment group. The treatment purpose was determined according to both the pretreatment examinations and the operation notes.

Follow-up schedules were established and applied referring to the NCCN Clinical Practice Guidelines. For patients who received curative treatment, they were followed up at out-patient department every three months for the first two years, then every six months for another three years and every one year for the rest of time. Patients with incurable disease continued to attend clinics or be hospitalized. For patients who didn’t follow the advice to come back to our hospital, we had a special follow-up department to make follow up telephone interviews.

### Statistical analysis

Differences of baseline and clinicopathological parameters between groups were evaluated by chi-square test, Mann–Whitney *U* test or Kruskal-Wallis H test based on the type of the data and comparison. Overall survival (OS) was the time interval from the date of diagnosis to death from ESCC or to the last date of follow-up. OS curves were plotted with the Kaplan-Meier method, and differences were compared with log-rank test. A Cox regression was used for univariate and multivariate analysis. Hazard ratio (HR) and 95% confidence interval (95% CI) were computed with the Cox proportional-hazards model. Variables significantly prognostic in univariate analysis were selected for multivariable analysis using the forward stepwise method. The optimal cutoff values for continuous prognostic indexes were determined with the method established by Jan Budczies *et al*. at http://molpath.charite.de/cutoff/ [15]. To evaluate the discriminatory ability of the inflammation-based prognostic scores, receiver operating characteristics (ROC) curves were generated, and the areas under the curve (AUC) were measured and compared. The statistical analyses were performed with SPSS 17.0 (SPSS Inc., Chicago, IL, USA). A two tailed *P* value <0.05 was considered statistically significant.

## Results

There were 423 patients pathologically confirmed with ESCC enrolled in this study. The median age was 58 years old, with an age range of 24 – 88 years old. The majority of patients were males (n = 341, (80.6%)). The numbers of patients from staged I to IV were 54 (12.8%), 168 (39.7%), 142 (33.6%), 59 (13.9%) respectively. Thirty six (8.5%) patients were with tumors located at upper esophagus, while there were 252 (59.6%) and 135 (31.9%) patients with tumors located at middle and lower esophagus respectively. There were 363 (85.8%) patients received tumor resection. The numbers of patients receiving curative and palliative treatment were 358 (84.6%) and 65 (15.2%) respectively.

The value of the CRP/Alb ratio ranged from 0.0 – 7.9 with a median of 0.055. The optimal cut-off value of the CRP/Alb ratio was determined to be 0.095 for the OS. We analyzed the association of the CRP/Alb ratio (≤0.095/> 0.095) with clinicopathologic characteristics of patients (Table 1). There was no difference in the distribution of age and sex between the two levels of the CRP/Alb ratio. However, it was found that a higher CRP/Alb ratio level (>0.095) was associated with more lymph node metastasis (*P* = 0.015), deeper tumor invasion (*P* = 0.003), more distant metastasis (*P* < 0.001) and more advanced TNM stage (*P* < 0.001). It, besides, was also associated with larger size of esophageal tumors (*P* < 0.001) and poorer tumor differentiation (*P* = 0.019). In addition, there were more patients without resection of esophageal tumors and received palliative treatment in the higher CRP/Alb ratio level group (both *P* < 0.001).

**Table 1:** **Correlation of the CRP/Alb ratio with the baseline and clinicopathological characteristics of patients**

Characteristic	No. of Patients (%)		*P*value
	CRP/Alb ≤ 0.095	CRP/Alb > 0.095	
Gender			0.093
Male	216 (78.3)	125 (85.0)	
Female	60 (21.7)	22 (15.0)	
Age (yr)	58 (24–88)	58 (24–78)	0.388
TNM stage (AJCC, 7th)			<0.001*
I	41 (14.9)	13 (8.8)	
II	131 (47.5)	37 (25.2)	
III	81 (29.3)	61 (41.5)	
IV	23 (8.3)	36 (24.5)	
N stage (AJCC, 7th)			0.015*
N0	154 (59.7)	49 (47.6)	
N1	58 (22.5)	23 (22.3)	
N2	35 (13.6)	25 (24.3)	
N3	11 (4.3)	6 (5.8)	
T stage (AJCC, 7th)			0.003*
T1	32 (12.3)	10 (8.7)	
T2	55 (21.1)	17 (14.8)	
T3	171 (65.5)	75 (65.2)	
T4	3 (1.1)	13 (11.3)	
M stage (AJCC, 7th)			< 0.001*
M0	253 (91.7)	111 (75.5)	
M1	23 (8.3)	36 (24.5)	
Primary tumor size (cm)	3.0 (0.5-11.0)	4.5 (1.0 -10.0)	< 0.001*
Tumor location			0.129
Upper	29 (10.5)	7 (4.8)	
Middle	160 (58.0)	92 (62.6)	
Lower	87 (31.5)	48 (32.7)	
Degree of differentiation			0.019*
Poorly or not differentiated	93 (33.7)	66 (44.9)	
Moderately differentiated	173 (62.7)	78 (53.1)	
Well differentiated	10 (3.6)	3 (2.0)	
Surgery			< 0.001*
No	18 (6.5)	42 (28.6)	
Yes	258 (93.5)	105 (71.4)	
Treatment purpose			< 0.001*
Curative treatment	256 (92.8)	102 (69.4)	
Palliative treatment	20 (7.2)	45 (30.6)	

*Significant differences between patients with the CRP/Alb ≤ 0.095 and patients with the CRP/Alb > 0.095.

*Abbreviation*: *TNM* tumor-node-metastasis, *AJCC* American Joint Committee on Cancer, *CRP/Alb* the C-reactive protein/Albumin ratio.

**Figure 1:**
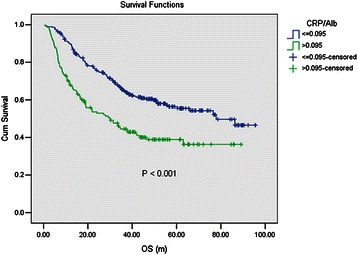
The prognostic value of the CRP/Alb ratio by univariate analysis. Compared with a lower CRP/Alb ratio (≤0.095), a higher CRP/Alb ratio (>0.095) was associated with significant worse OS (*P* < 0.001).

The median follow-up time was 35.7 months, with a range of 0.6 – 95.6 months. The median OS was 60.5 months for the whole cohort of patients. Compared with a lower CRP/Alb ratio (≤0.095), a higher CRP/Alb ratio (>0.095) was associated with significant worse OS (*P* < 0.001, Figure 1). Other significant prognostic indexes identified by univariate analysis included age (≤54/> 54 yr), the TNM stage (I/II/III/IV), distant metastasis (No/Yes), surgery (No/Yes), treatment purpose (Curative treatment/Palliative treatment), BMI (≤20.43/> 20.43), mGPS (0/1/2), PNI (≤49.05/> 49.05), NLR (≤1.835/> 1.835), PLR (≤163.8/> 163.8), CRP (≤10/> 10), white blood cell (WBC) (≤10/> 10), albumin (≤35/> 35). The detailed results were shown in Table 2.

**Table 2:** **Prognostic factors for overall survival identified by univariate and multivariate analyses**

Characteristics	Univariate analysis		Multivariate analysis		
	No. (%)	*p*value	Hazard ratio	95% CI	*P*value
Sex		0.766			
Male	341 (80.6)				
Female	82 (19.4)				
Age (yr)		0.013*	1.473	1.079 - 2.012	0.015*
≤54	146 (34.5)				
>54	277 (65.5)				
Tumor location		0.485			
Upper	36 (8.5)				
Middle	252 (59.6)				
Lower	135 (31.9)				
Degree of differentiation		0.167			
Poorly or not differentiated	159 (37.6)				
Moderately differentiated	251 (59.3)				
Poorly differentiated	13 (3.1)				
TNM stage (AJCC, 7th)		<0.001*			<0.001*
I	54 (12.8)		1	Reference	
II	168 (39.7)		1.913	0.973 – 3.760	0.060
III	142 (33.6)		4.231	2.178 – 8.220	<0.001*
IV	59 (13.9)		3.552	1.405 – 8.978	0.007*
Distant metastasis		<0.001*			
No	364 (86.1)				
Yes	59 (13.9)				
Surgery		<0.001*			
No	60 (14.2)				
Yes	363 (85.8)				
Treatment purpose		<0.001*	2.113	1.099 – 4.061	0.025*
Curative treatment	358 (84.6)				
Palliative treatment	65 (15.2)				
BMI (Kg/m^2^)		<0.001*	0.663	0.496 – 0.885	0.005*
≤20.43	158 (37.4)				
>20.43	257 (60.8)				
CRP/Alb		<0.001*	1.393	1.031 – 1.883	0.031*
≤0.095	276 (65.2)				
>0.095	147 (34.8)				
mGPS		0.003*			
0	345 (81.6)				
1	70 (16.5)				
2	8 (1.9)				
PNI		0.002*			
≤49.05	102 (24.1)				
>49.05	321 (75.9)				
NLR		0.018*			
≤1.835	139 (32.9)				
>1.835	284 (67.1)				
PLR		0.026*			
≤163.8	327 (77.3)				
>163.8	96 (22.7)				
CRP (mg/L)		0.002*			
≤10	345 (81.6)				
>10	78 (18.4)				
WBC (×10^9/L)		0.365			
≤10	373 (88.2)				
>10	50 (11.8)				
Albumin (g/L)		0.041*			
≤35	8 (1.9)				
>35	415 (98.1)				

*Statistically significant prognostic factor identified by univariate/multivariate analysis.

*Abbreviation*: *CI* confidence interval, *TNM* tumor-node-metastasis, *AJCC* American Joint Committee on Cancer, *BMI* body mass index, *CRP/Alb* the C-reactive protein/Albumin ratio, *mGPS* the modified Glasgow Prognostic Score, *PNI* the prognostic nutritional index, *NLR* the neutrophil lymphocyte ratio, *PLR* the platelet lymphocyte ratio, *CRP* C-reactive protein, *WBC* white blood cell.

The cut-off values for age, BMI, CRP/Alb, PNI, NLR and PLR were determined by the method described in statistic analysis. CRP, WBC and Albumin were categorized according to clinical normal reference range.

These variables were selected for multivariate analysis using a forward stepwise method, and five indexes were identified to be independent prognostic factors for OS. They were age (*HR* 1.473, *P* = 0.015), the TNM stage (*P* < 0.001), treatment purpose (*HR* 2.113, *P* = 0.025), BMI (*HR* 0.663, *P* = 0.005) and the CRP/Alb ratio (*HR* 1.393, *P* = 0.031).

In order to further identify features of patients with better value of the CRP/Alb ratio for prognostic application in ESCC, we performed subgroup survival analysis. The results were presented in Additional file 1: Table S1. It was found that the CRP/Alb ratio remained to be a significant prognostic factor in all subgroups except female patients, patients with upper esophageal tumors and patients with curative treatment. However, in multivariate analysis, its prognostic value remained significant in partial patients, including male patients, patients at younger age (≤54), patients with moderately differentiated tumors, patients with distant metastasis, patients without esophageal tumor resection and patients with palliative treatment.

**Figure 2:**
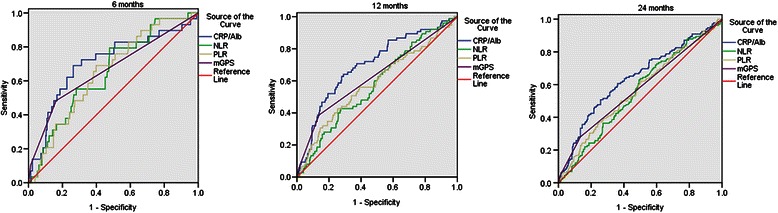
The ROC curves of inflammation-based prognostic indexes at 6, 12 and 24 months of follow-up. This figure showed the ROC curves of the CRP/Alb ratio (continuous), NLR (continuous), PLR (continuous) and mGPS (categorical) for the survival status at 6 months, 1 year and 2 years of follow-up.

To compare the discriminatory ability of the CRP/Alb ratio, a novel inflammation-based prognostic score with that of other established inflammation-based prognostic indexes, we generated ROC curves for the survival status at 6 months, 1 year and 2 years of follow-up and statistically compared the differences of estimated AUC (Table 3). It was found that at the follow-up of 1 year, the AUC value of the CRP/Alb ratio was significantly higher than that of the NLR and PLR. At the follow-up of 2 years, the AUC value of CRP/Alb ratio remained to be significantly higher than that of NLR. No significant difference of AUC value was found between the CRP/Alb ratio and mGPS. In addition, we found that along with the extension of follow-up time, the TNM stage remained to have higher discriminatory ability, while all the inflammation-based scores showed decreased discriminatory ability. The detailed information was demonstrated in Table 3. The ROC curves of the inflammation-based prognostic indexes were shown in Figure 2.

**Table 3:** **Comparisons of the discriminatory ability of prognostic scores by comparing the AUC**

Indexes	AUC	95% CI	*P*value	Significance of comparison §*P’*value
6 months of follow-up				
TNM stage (AJCC, 7th)	0.752	0.658 – 0.847	<0.001*	0.53
CRP/Alb (continuous)	0.706	0.597 – 0.815	<0.001*	-
CRP/Alb (categorical)	0.702	0.604 – 0.800	<0.001*	-
mGPS	0.665	0.550 – 0.781	0.003*	0.61
NLR (continuous)	0.649	0.553 – 0.745	0.007*	0.44
NLR (categorical)	0.584	0.484 – 0.683	0.132	0.09
PLR (continuous)	0.650	0.557 – 0.743	0.007*	0.45
PLR (categorical)	0.563	0.450 – 0.677	0.255	0.07
1 year of follow-up				-
TNM stage (AJCC, 7th)	0.706	0.642 – 0.771	<0.001*	0.87
CRP/Alb (continuous)	0.698	0.631 – 0.765	<0.001*	-
CRP/Alb (categorical)	0.670	0.600 – 0.739	<0.001*	-
mGPS	0.626	0.550 – 0.701	0.001*	0.16
NLR (continuous)	0.576	0.506 – 0.645	0.040*	0.01*
NLR (categorical)	0.552	0.482 – 0.621	0.162	0.02*
PLR (continuous)	0.584	0.509 – 0.659	0.022*	0.03*
PLR (categorical)	0.574	0.499 – 0.648	0.046*	0.06
2 years of follow-up				
TNM stage (AJCC, 7th)	0.731	0.680 – 0.783	<0.001*	0.02*
CRP/Alb (continuous)	0.638	0.579 – 0.697	<0.001*	-
CRP/Alb (categorical)	0.619	0.560 – 0.679	<0.001*	-
mGPS	0.574	0.513 – 0.636	0.015*	0.14
NLR (continuous)	0.555	0.496 – 0.614	0.074	0.05*
NLR (categorical)	0.550	0.491 – 0.609	0.102	0.10
PLR (continuous)	0.566	0.506 – 0.626	0.031*	0.09
PLR (categorical)	0.556	0.495 – 0.617	0.067	0.14

*Significant *P* value of ROC curves analysis.

*Abbreviation*: *ROC* receiver operating characteristics, *AUC* area under the curves, *TNM* tumor-node-metastasis, *AJCC* American Joint Committee on Cancer, *CRP/Alb* the C-reactive protein/Albumin ratio, *mGPS* the modified Glasgow Prognostic Score, *NLR* the neutrophil lymphocyte ratio, *PLR* the platelet lymphocyte ratio.

§ Comparisons of AUC values were made between the CRP/Alb ratio and other inflammation-based prognostic factors using z test method, a two tailed *P* value <0.05 was considered statistically significant. Significant differences were marked with “*”. Continuous indexes were compared with the CRP/Alb ratio as a continuous variable, while categorical indexes were compared with the CRP/Alb ratio as a categorical variable.

We also explored the association of the CRP/Alb ratio (≤0.095/> 0.095) with other established nutrition and inflammation-based prognostic indexes, including the mGPS, PNI, NLR, PLR, CRP, WBC, albumin and BMI (Table 4). It was found that the CRP/Alb ratio was associated with all these indexes (all *P* < 0.001, except for *P* = 0.001 for BMI). Interestingly, in terms of the mGPS score, most of the patients (n = 345, 81.6%) were classified into the group of score 0, and they were classified by the CRP/Alb ratio (≤0.095/> 0.095) into two groups. However, no patients with mGPS score of 1 and 2 had the CRP/Alb ratio ≤ 0.095 (Table 4).

**Table 4:** **Correlation of the CRP/Alb ratio with other nutrition and/or inflammation-based prognostic scores**

Characteristic	No. of Patients (%)		*P*value
	CRP/Alb ≤ 0.095	CRP/Alb > 0.095	
mGPS			<0.001*
0	276 (100)	69 (46.9)	
1	0	70 (47.6)	
2	0	8 (5.4)	
PNI	53.8 (41.9 - 70.6)	50.4 (33.5 - 59.6)	<0.001*
NLR	1.9 (0.5 - 28.0)	3.2 (1.2 - 27.7)	<0.001*
PLR	111.5 (0.8 – 990.0)	147.1 (55.2 – 830.0)	<0.001*
CRP (mg/L)	1.2 (0.1 – 4.5)	10.6 (3.9 – 233.0)	<0.001*
WBC (×10^9/L)	6.7 (1.7 – 15.7)	8.4 (3.8 – 23.9)	<0.001*
Albumin (g/L)	43.8 (36.1 – 54.6)	41.7 (29.5 – 48.6)	<0.001*
BMI (Kg/ m^2^)	21.5 (14.9 – 30.1)	20.6 (13.4 – 32.2)	0.001*

*Statistically significant differences of distribution between patients with the CRP/Alb ratio ≤ 0.095 and patients with the CRP/Alb ratio > 0.095.

*Abbreviation*: *CRP/Alb* the C-reactive protein/Albumin ratio, *mGPS* the modified Glasgow Prognostic Score, *PNI* the prognostic nutritional index, *NLR* the neutrophil lymphocyte ratio, *PLR* the platelet lymphocyte ratio, *CRP* C-reactive protein, *WBC* white blood cell, *BMI* body mass index.

**Figure 3:**
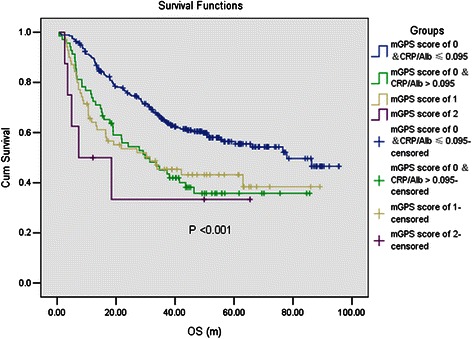
The prognostic value by Kaplan-Meier curves of the combination of the CRP/Alb ratio and mGPS. All patients were classified into four groups according to the mGPS score and CRP/Alb level: mGPS score of 0 &CRP/Alb ≤ 0.095, mGPS score of 0 & CRP/Alb > 0.095, mGPS score of 1 and mGPS score of 2. Patients with a mGPS score of 0&CRP/Alb ≤ 0.095 had the best overall survival, while patients with a mGPS score of 0 & CRP/Alb > 0.095 and a mGPS score of 1 had comparable moderate overall survival, and patients with a mGPS score of 2 had the worst overall survival.

We further categorized patients into four groups according to the mGPS score and CRP/Alb ratio level: mGPS score of 0 &CRP/Alb ratio ≤ 0.095, mGPS score of 0 & CRP/Alb ratio > 0.095, mGPS score of 1 and mGPS score of 2. The Kaplan-Meier curves were shown in Figure 3 (*P* <0.001). Patients with mGPS score of 0&CRP/Alb ≤ 0.095 had the best OS, while patients with mGPS score of 0 & CRP/Alb > 0.095 and mGPS score of 1 had comparable moderate OS, and patients with mGPS score of 2 had the worst OS.

## Discussion

It has been recognized that inflammation is an important regulator in the genesis, progression and metastasis of malignancies [16,17]. Inflammatory factors derive not only from the systemic reaction to malignancies, but also the secretion of tumor cells, including acute phase proteins like CRP, [18,19] chemokines [20], cytokines like interleukin 6 (IL-6) [21], transcription factor like NF-κB [22], circulating and infiltration immune cells [23] and so on. The host factors and tumor factors interact with each other, causing some systemic symptoms, such as pyrexia and cachexia. Their interactions can also accelerate tumor progression or result in tumor regression. Thus the levels of inflammatory components have certain prognostic value in cancer, and this theory has been demonstrated by extensive studies [12,14,24,25]. In addition, malnutrition is correlated with poor performance status and worse survival [26]. To predict survival of cancer patients expediently, previous researches have established some nutrition and inflammation-based indexes derived from routine tests, such as CRP, mGPS, PNI, NLR, PLR, and Albumin.

The CRP/Alb ratio was primarily developed to identify patients with serious illness on an acute medical ward by Fairclough, E *et al*. [27]. Then another study assessed its ability to predict 90-day mortality of septic patients [28]. More recently, Kinoshita, A *et al*. explored its prognostic value in hepatocellular carcinoma. They found that it had comparable performance with mGPS and better performance than NLR [14]. Our study assessed the clinicopathologic relevance and prognostic value of the CRP/Alb ratio in ESCC. We found that it had significant association with some important clinicopathologic characteristics. In univariate analysis, all of the inflammation-based prognostic indexes were found to be significant prognostic. However, after adjusting for confounding factors, only the CRP/Alb ratio remained to be a significant prognostic factor. Besides, compared with the NLR and PLR, the CRP/Alb ratio had better discriminatory ability. What’s more, the CRP/Alb ratio was significantly associated with all these inflammation-based prognostic indexes, suggesting that it might absorb the prognostic value of all those indexes and had a combined predictive effect. In general, these results indicated that the CRP/Alb ratio was a novel and promising inflammation-based prognostic score in ESCC.

The the CRP/Alb ratio and mGPS were both calculated with the same indexes, CRP and albumin. Since the mGPS was one of the best acknowledged and demonstrated inflammation-based prognostic scores in varieties of cancer scenarios [11], it drew special attention to compare the prognostic value of the two prognostic scores. Although the multivariate analysis showed that the CRP/Alb ratio was an independent prognostic factor for OS while the mGPS was a confounding factor, the comparison of AUC value identified no differences in the discriminatory ability between the two prognostic scores. What further caught our attention was that when classified by the mGPS, there were 81.6% of patients classified in the group of a score of 0, which meant that the mGPS couldn’t distinguish the survival differences of most of the patients in this study. This was in consistent with some previous researches in EC [9,29]. We suggested that the combination of the mGPS and CRP/Alb ratio would better distinguish the survival differences of cancer patients. Thus we categorized patients into four groups and explored their survival differences: mGPS score of 0 &CRP/Alb ≤ 0.095, mGPS score of 0 & CRP/Alb > 0.095, mGPS score of 1 and mGPS score of 2. It could be found that ARP/Alb identified a group of patients with mGPS score of 0 to have comparable survival with mGPS score of 1. As a continuous score, the CRP/Alb might have the ability to identify tiny differences among patients classified into the same group by the mGPS score. Therefore the CRP/Alb would be a significant coadjutant for the mGPS to predict survival.

In this study, a higher CRP/Alb ratio was associated with lower BMI level and albumin level (Table 4). As a matter of fact, the association between inflammation and nutrition had been comfirmed by plenty of researches, and systemic inflammatory response was found to be related to poor performance status, nutritional decline and subsequent poor outcome in cancer patients [30-33]. Several studies proved that supplement of some trophic factors, for example, ω-3 polyunsaturated fatty acids, could improve plasma fatty acid profile, CRP/Alb status, and immune function and prevent weight loss during treatment in cancer patients [34,35]. Since CRP/Alb ratio was related with not only other inflammation-based indexes, but also nutrition-based indexes, as well as both short and long-term outcomes of patients, it would probably be an appropriate index to evaluate and predict the effectiveness of nutrition improvement treatment of cancer patients in clinical practice.

Our study indicated that the CRP/Alb level was associated with aggressive behavior. The mechanism of how inflammation regulates tumor behavior and host status was complicated. The production of CRP was independently mediated by IL-6 level [36]. This way of regulation was partially responsible for poor response to chemoradiotherapy in patients with EC [37]. IL-6 also stimulated recruitment of myeloid-derived suppressor cells, and induced invasive tumor in ESCC [38]. STAT3 and NF-κB could be activated by IL-6 to prevent apoptosis and promote proliferation of malignant tumor cells [16]. In addition, the phenotype of infiltrating immune cells in the tumor microenvironment was mediated by cytokines, chemokines and other inflammatory mediators [16]. And immune cell infiltration was a prognostic marker in ESCC [39]. Mesenchymal stem cells were also found to have some interactions with inflammation in tumor microenvironment [40]. Above were the examples of mechanism of inflammation-related tumor invasive characteristics. Cachexia was a common and devastating symptom in malignancies. It was induced by metabolic dysfunction resulted from complex crosstalk of inflammatory cytokines. Other symptoms such as fever, weight loss and fatigue were all associated with higher concentrations of certain inflammatory factors [41]. However, there was still some room for more and deepened researches, and therapeutic application directing at malignant inflammation was promising.

The main limitation of our study was that it was conducted retrospectively in a single center, and the prognostic value of the CRP/Alb ratio was not verified in a validation cohort. Besides, there was large heterogeneity in the patient treatment, thus it was hard to analyze the impact of the CRP/Alb ratio on patients outcome in different treatment patterns. In addition, compared with the mGPS, the continuity of the CRP/Alb ratio required an optimal cut-off value in clinical practice. However, it might be feasible to find an optimal cut-off value for each tumor stage. In conclusion, our study demonstrated the CRP/Alb ratio to be a promising inflammation-based prognostic score. It was significantly associated with more invasive clinicopathologic characteristics and worse patient outcomes in ESCC. It was superior to some established inflammation-based prognostic indexes, including the NLR and PLR. Last but not least, it was a valuable coadjutant for the mGPS to predict OS of patients with ESCC. The prognostic value of the CRP/Alb ratio should be further evaluated in larger prospective studies and other malignancies.

## Conclusions

Our study demonstrated that the CRP/Alb ratio was associated with some important clinicopathological characteristics in ESCC, including tumor size (*P* < 0.001), tumor differentiation (*P* = 0.019), T stage (*P* = 0.003), N stage (*P* = 0.015), M stage (*P* < 0.001) and TNM stage (*P* < 0.001). In addition, a higher CRP/Alb ratio was associated with worse OS (*P* = 0.031 by multivariate analysis). Compared with the NLR and PLR, the CRP/Alb ratio showed a superior discriminatory ability. Although no statistical difference of discriminatory ability was found between the CRP/Alb ratio and the mGPS, the CRP/Alb ratio could identify a group of patients with mGPS score of 0, who had comparable overall survival with those with mGPS score of 1 (*P* < 0.001). In conclusion, our study demonstrated the CRP/Alb ratio to be a novel and promising prognostic inflammation-based factor in ESCC.

## Additional file

Additional file 1: Table S1. Prognostic value the CRP/Alb ratio for overall survival in subgroups by univariate and multivariate analyses.

## Abbreviation

CRP: C-reactive protein
CRP/Alb ratio: C-reactive protein/albumin ratio
AUC: Area under the curves
mGPS: the modified Glasgow Prognostic Score
PNI: Prognostic nutritional index
NLR: Neutrophil lymphocyte ratio
PLR: Platelet lymphocyte ratio
EC: Esophageal cancer
EAC: Esophageal adenocarcinoma
ESCC: Esophageal squamous cell carcinoma
AJCC: the American Joint Committee on Cancer
UICC: the Union for International Cancer Control
TNM: Tumor-node-metastasis
BMI: Body mass index
NCCN: the National Comprehensive Cancer Network
OS: Overall survival
HR: Hazard ratio
CI: Confidence interval
ROC: Receiver operating characteristics
WBC: White blood cell
IL-6: Interleukin 6
