# Supplementary material for: A novel inflammation-based prognostic score in esophageal squamous cell carcinoma: the C-reactive protein/albumin ratio
Source: BMC Cancer. 2015 May 2;15:350. doi: 10.1186/s12885-015-1379-6 (PMC4423167; doi:10.1186/s12885-015-1379-6)
Supplement: Additional file 1: Table S1. — Prognostic value the CRP/Alb ratio for overall survival in subgroups by univariate and multivariate analyses. [file 12885_2015_1379_MOESM1_ESM.docx]

Additional file 1: Table S1 Prognostic value the CRP/Alb ratio for overall survival in subgroups by univariate and multivariate analyses.

| subgroups | Univariate analysis | | Multivariate analysis | |
| --- | --- | --- | --- | --- |
|  | No. (%) | *p* value | Hazard ratio and 95% CI | *p* value |
| Sex |  |  |  |  |
| Male | 341 | < 0.001* | 1.48 (1.06 – 2.07) | 0.02* |
| Female | 82 | 0.26 | - | - |
| Age (yr) |  |  |  |  |
| ≤ 54 | 146 | < 0.001* | 3.06 (1.77 – 5.28) | < 0.001* |
| > 54 | 277 | 0.012* | - | - |
| Tumor location |  |  |  |  |
| Upper | 36 | 0.21 | - | - |
| Middle | 252 | 0.001* | - | - |
| Lower | 135 | 0.003* | - | - |
| Degree of differentiation |  |  |  |  |
| Poorly or not differentiated | 159 | 0.013* | - | - |
| Moderately differentiated | 251 | 0.001* | 1.49 (1.00 – 2.22) | 0.05* |
| Well differentiated | 13 | 0.001* | - | - |
| Tumor stage (AJCC, 7th) |  |  |  |  |
| I + II + III | 364 | 0.01* | - | - |
| IV | 59 | 0.006* | 2.32 (1.19 – 4.56) | 0.01* |
| Surgery |  |  |  |  |
| No | 60 | 0.02* | 2.16 (1.06 – 4.43) | 0.04* |
| Yes | 363 | 0.03* |  |  |
| Treatment purpose |  |  |  |  |
| Curative treatment | 358 | 0.08 | - | - |
| Palliative treatment | 65 | 0.003* | 2.49 (1.22 – 5.07) | 0.01* |

* Statistically significant prognostic factor identified by univariate/multivariate analysis.

Abbreviation: CI, confidence interval; TNM, tumor-node-metastasis; AJCC, American Joint Committee on Cancer; CRP/Alb, the C-reactive protein/Albumin ratio;
